# Supplementary material for: Soil community composition in dynamic stages of semi-natural calcareous grassland
Source: PLoS One. 2023 Oct 17;18(10):e0292425. doi: 10.1371/journal.pone.0292425 (PMC10581465; doi:10.1371/journal.pone.0292425)
Supplement: S1 Table — (PDF) [file pone.0292425.s001.pdf]

	Site	Unmanaged open	Managed open	Managed previous scrub	Managed previous forest
1	Aruküla	Pr / F / Pl	Pr / F / Pl	Pr / F / Pl	Pr / F / Pl
2	Asva1	Pr / F / Pl	Pr / F / Pl	Pr / F / Pl	Pr / F / Pl
3	Eeriksaare	Pr / F / Pl	Pr / F / Pl	Pr / F / Pl	Pr / F / Pl
4	Heltermaa lisa	Pr / F / Pl	Pr / Pl	Pr / Pl	Pr / F / Pl
5	Ilpla	Pr / Pl	Pr / Pl	Pr / Pl	Pr / Pl
6	Kahtla1	Pr / F / Pl	Pr / Pl	Pr / F / Pl	Pr / F / Pl
7	Kahtla2	Pr / Pl	Pr / F / Pl	Pr / F / Pl	Pr / Pl
8	Kassari	Pr / Pl	Pl	Pr / F / Pl	Pr / F / Pl
9	Koguva		Pr / F / Pl	Pr / Pl	Pr / F / Pl
10	Kõruse	Pr / Pl	Pr / Pl	Pr / Pl	Pr / Pl
11	Kurese	Pr / Pl	Pr / F / Pl	Pr / Pl	Pr / F / Pl
12	Lõetsa1	Pr / F / Pl	Pr / Pl	Pr / Pl	Pr / Pl
13	Lõetsa2		Pr / F / Pl	Pr / F / Pl	Pr / F / Pl
14	Lõu	Pr / F / Pl	Pr / Pl	Pr / Pl	Pr / Pl
15	Neeme	Pr / Pl	Pr / F / Pl	Pr / F / Pl	Pr / Pl
16	Nõmmküla	Pr / Pl	Pr / Pl	Pr / Pl	Pr / Pl
17	Paadla	Pr / Pl	F / Pl	Pr / F / Pl	Pr / F / Pl
18	Paenase	Pr / F / Pl	Pr / F / Pl	Pr / F / Pl	Pr / Pl
19	Paope	Pr / Pl	Pr / F / Pl	Pr / F / Pl	Pr / Pl
20	Sarve		Pr / F / Pl	Pr / F / Pl	Pr / F / Pl
21	Tammese	Pr / Pl	Pr / F / Pl	Pr / F / Pl	Pr / F / Pl
22	Türju	Pr / Pl	Pr / Pl	Pr / Pl	
23	Üügu	Pr / Pl	Pr / Pl	Pr / Pl	
24	Vanamõisa	Pr / F / Pl	Pr / Pl	Pr / Pl	Pr / F / Pl
25	Virtsu	Pr / F / Pl	Pr / F / Pl	Pr / F / Pl	
26	Vohilaiu		F / Pl		Pr / Pl
27	Võiküla1	Pr / F / Pl	Pr / Pl	Pr / F / Pl	Pr / F / Pl
28	Võiküla3		F / Pl	F / Pl	
29	Võrsna	Pr / Pl	Pr / Pl	Pr / Pl	Pr / F / Pl

Pr – Prokaryotes
F – Fungi
Pl - Plants

Total number of sites = 29
Total number of subsites sites expected = 116
Missing samples = 10
Number of subsites used for the analysis = 106
