# Supplementary figures and images for: Soil community composition in dynamic stages of semi-natural calcareous grassland
Source: PLoS One. 2023 Oct 17;18(10):e0292425. doi: 10.1371/journal.pone.0292425 (PMC10581465; doi:10.1371/journal.pone.0292425)

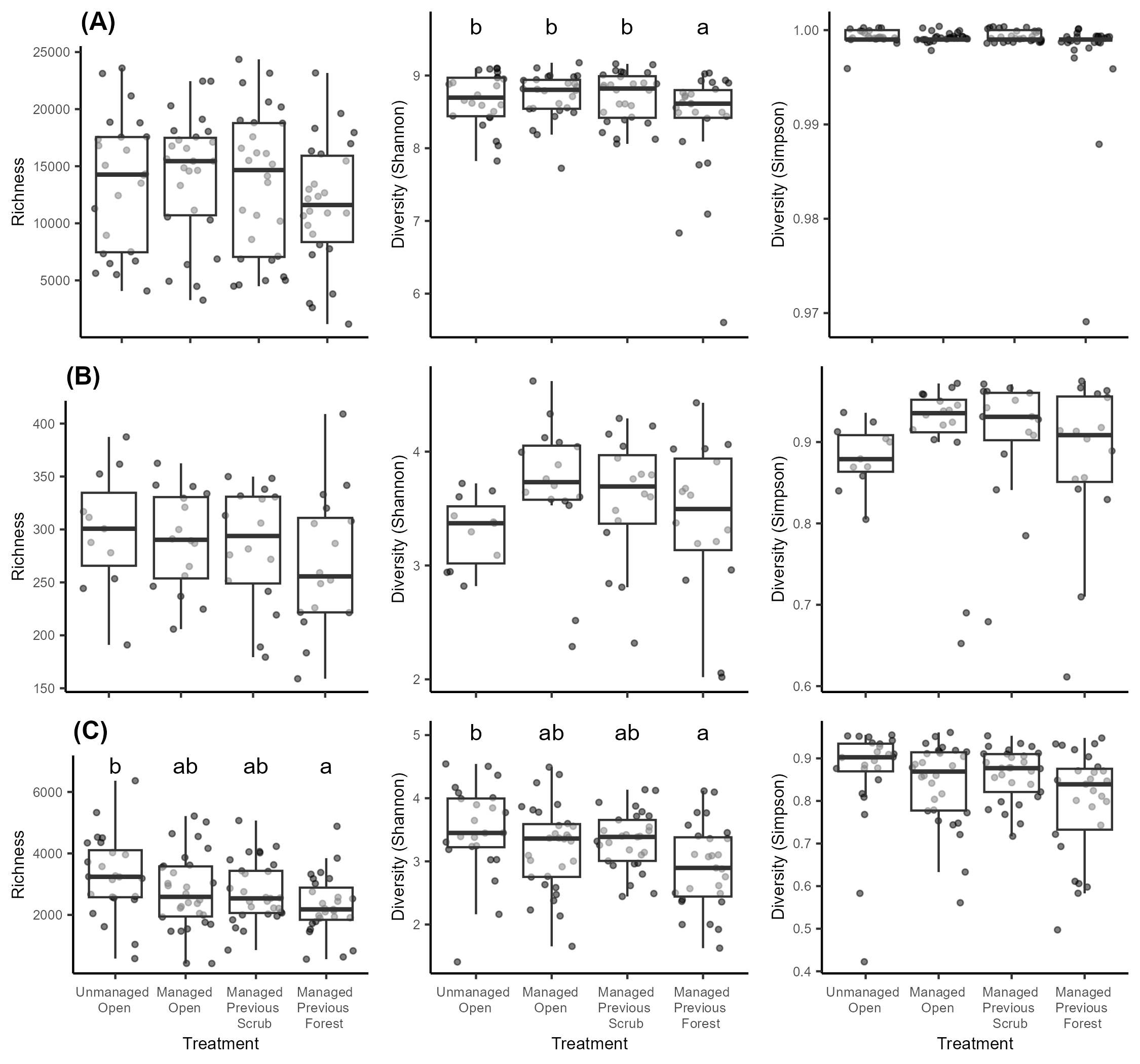

Supplement: S1 Fig — The letters above the boxes indicate significant differences between groups. (TIF) [file pone.0292425.s002.tif]

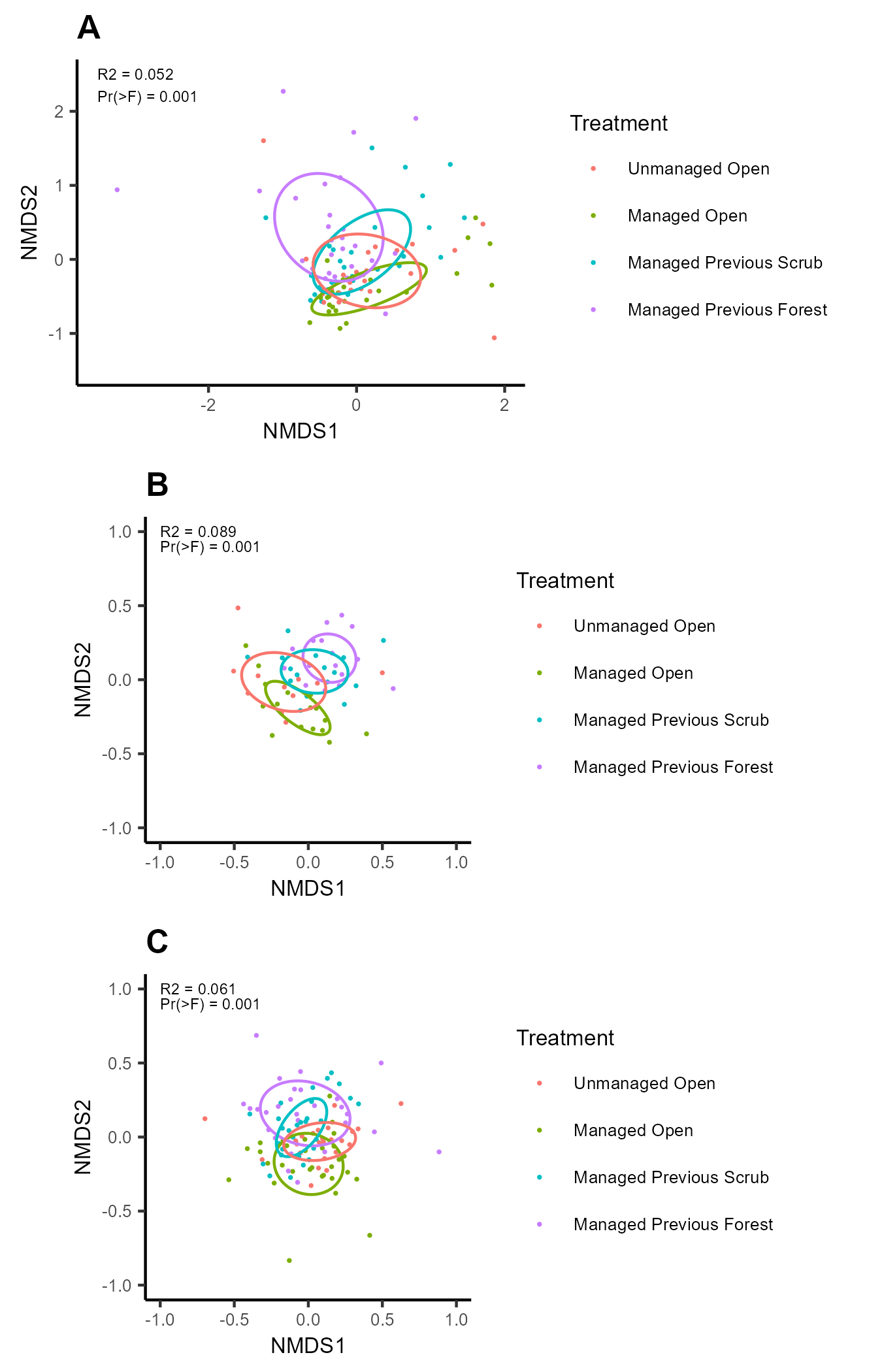

Supplement: S2 Fig — (TIF) [file pone.0292425.s003.tif]

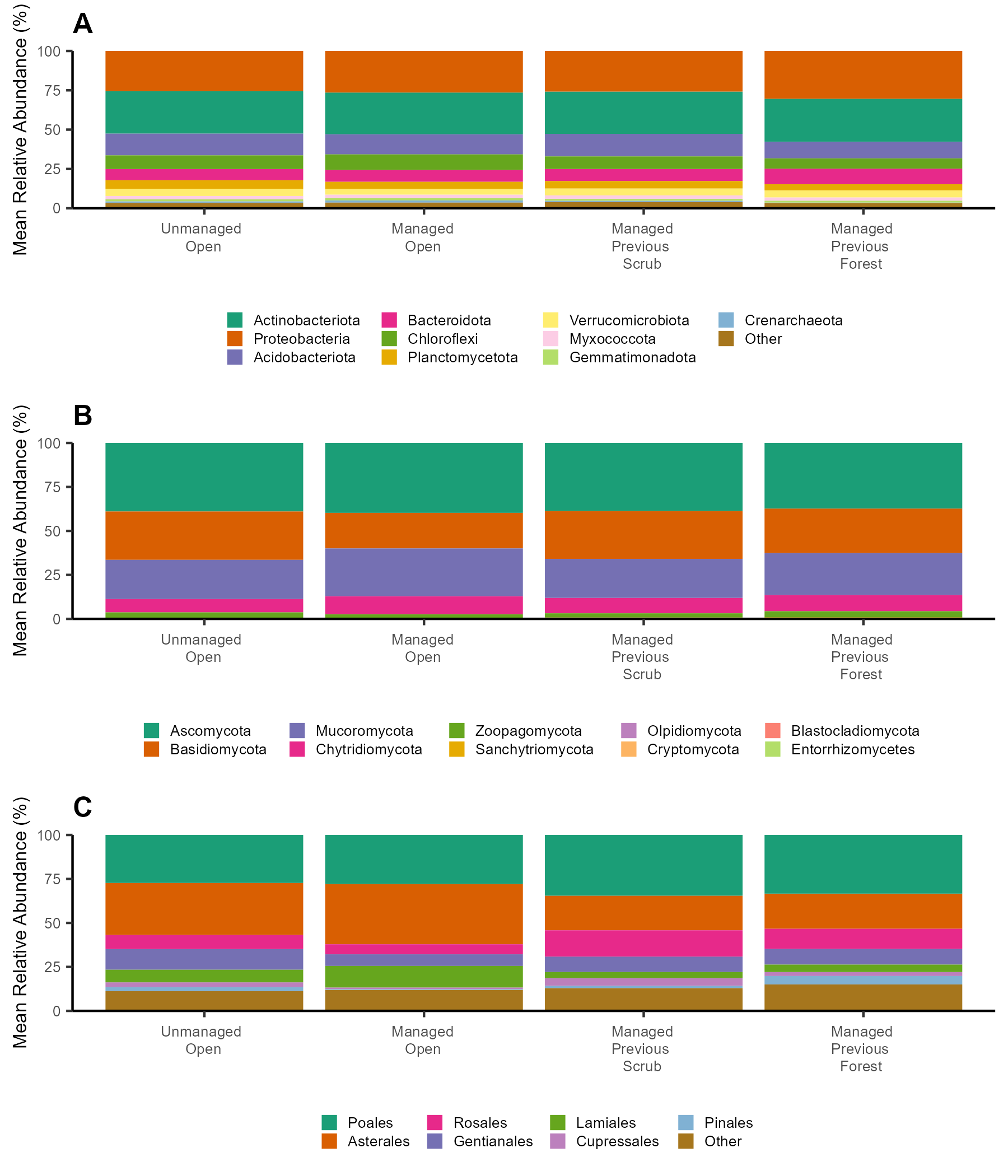

Supplement: S3 Fig — (TIF) [file pone.0292425.s004.tif]

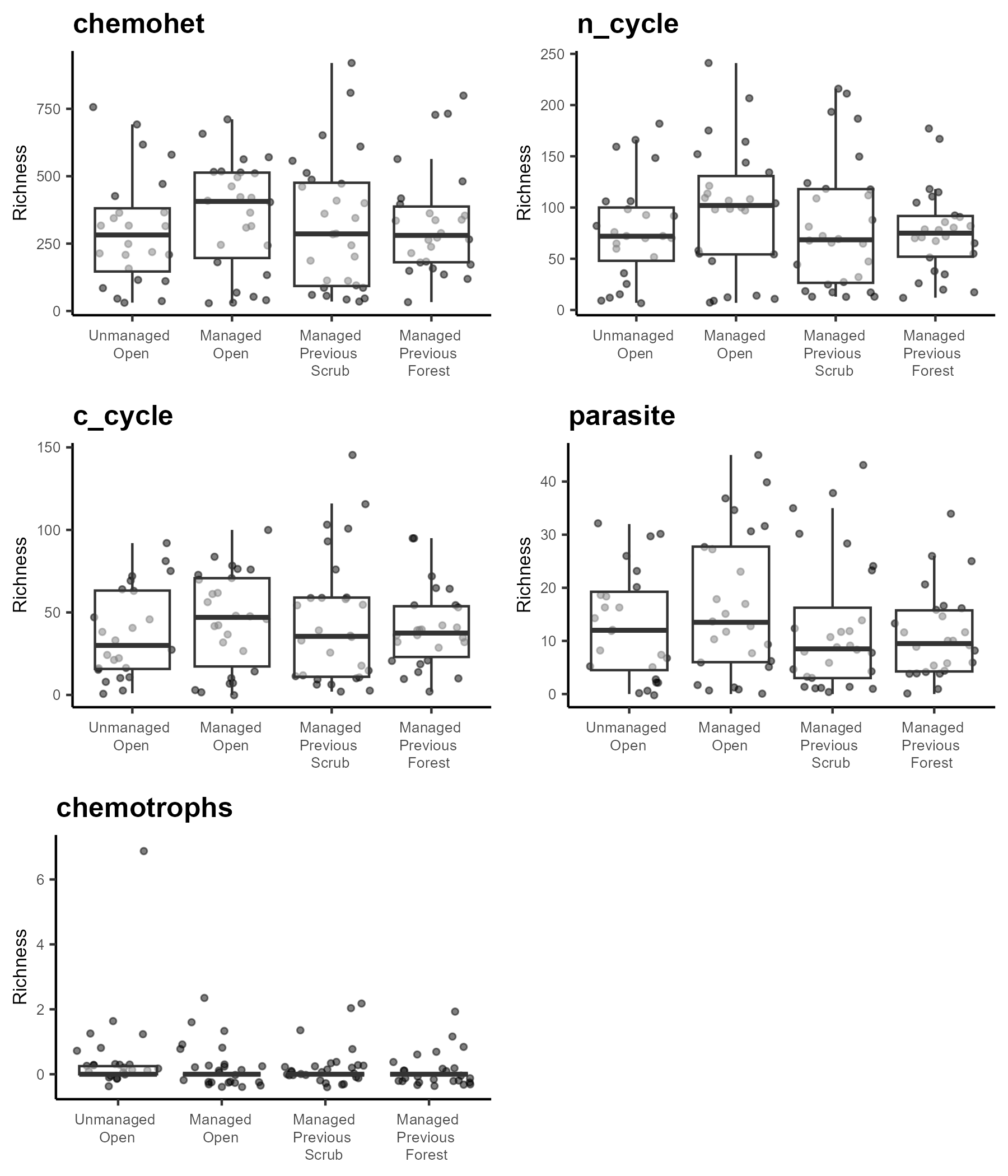

Supplement: S4 Fig — (TIF) [file pone.0292425.s005.tif]

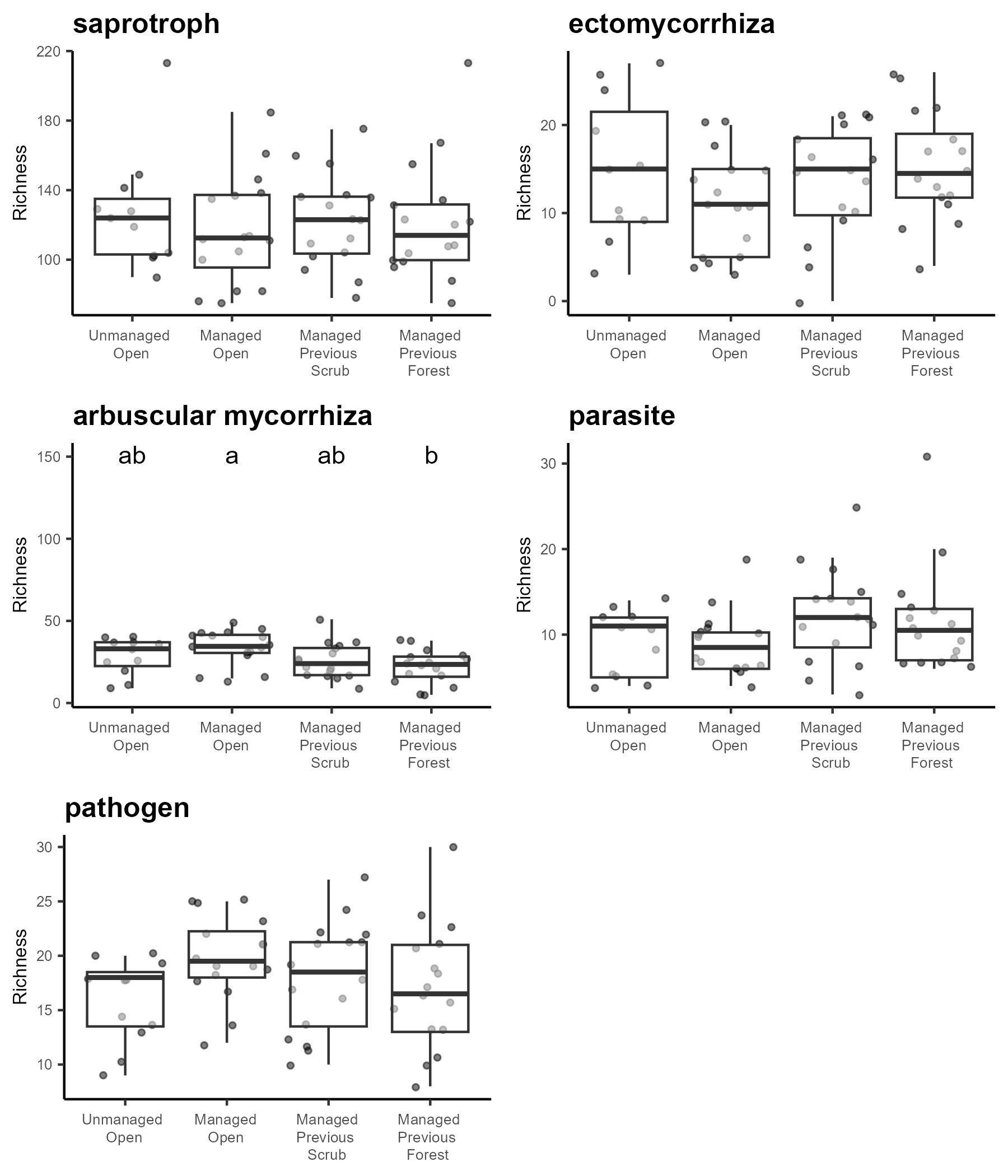

Supplement: S5 Fig — The letters above the boxes indicate significant differences between groups. (TIF) [file pone.0292425.s006.tif]

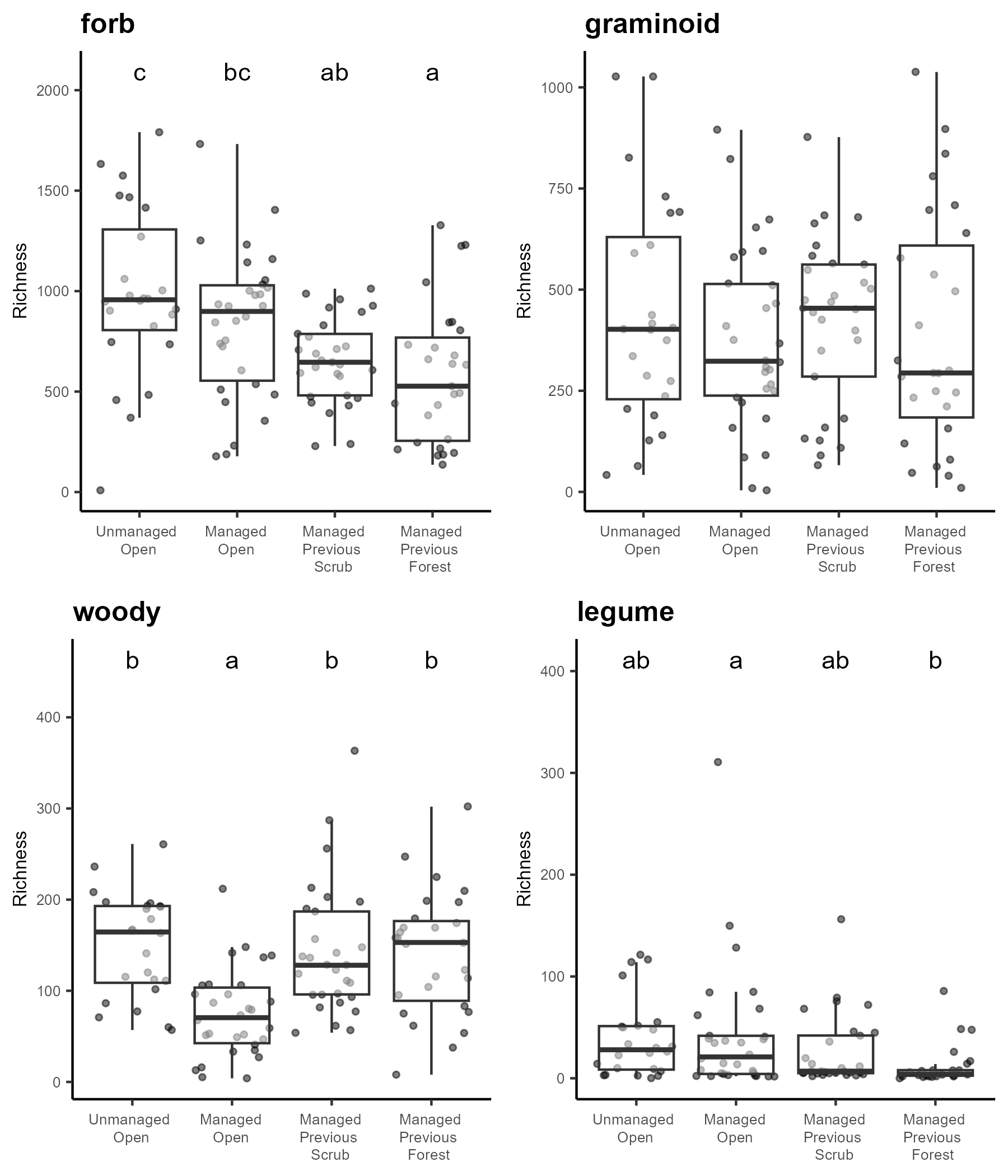

Supplement: S6 Fig — The letters above the boxes indicate significant differences between groups. (TIF) [file pone.0292425.s007.tif]

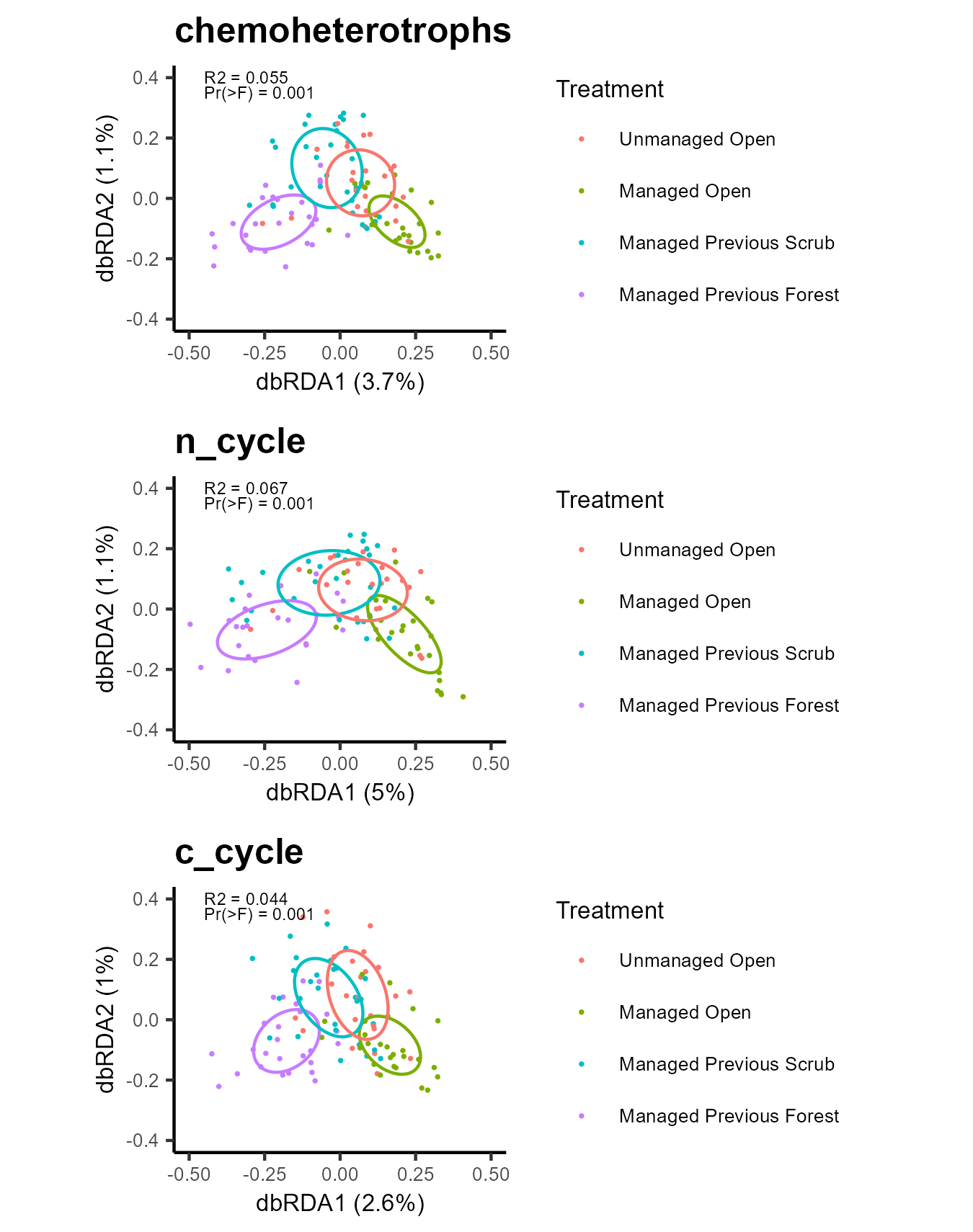

Supplement: S7 Fig — (TIF) [file pone.0292425.s008.tif]

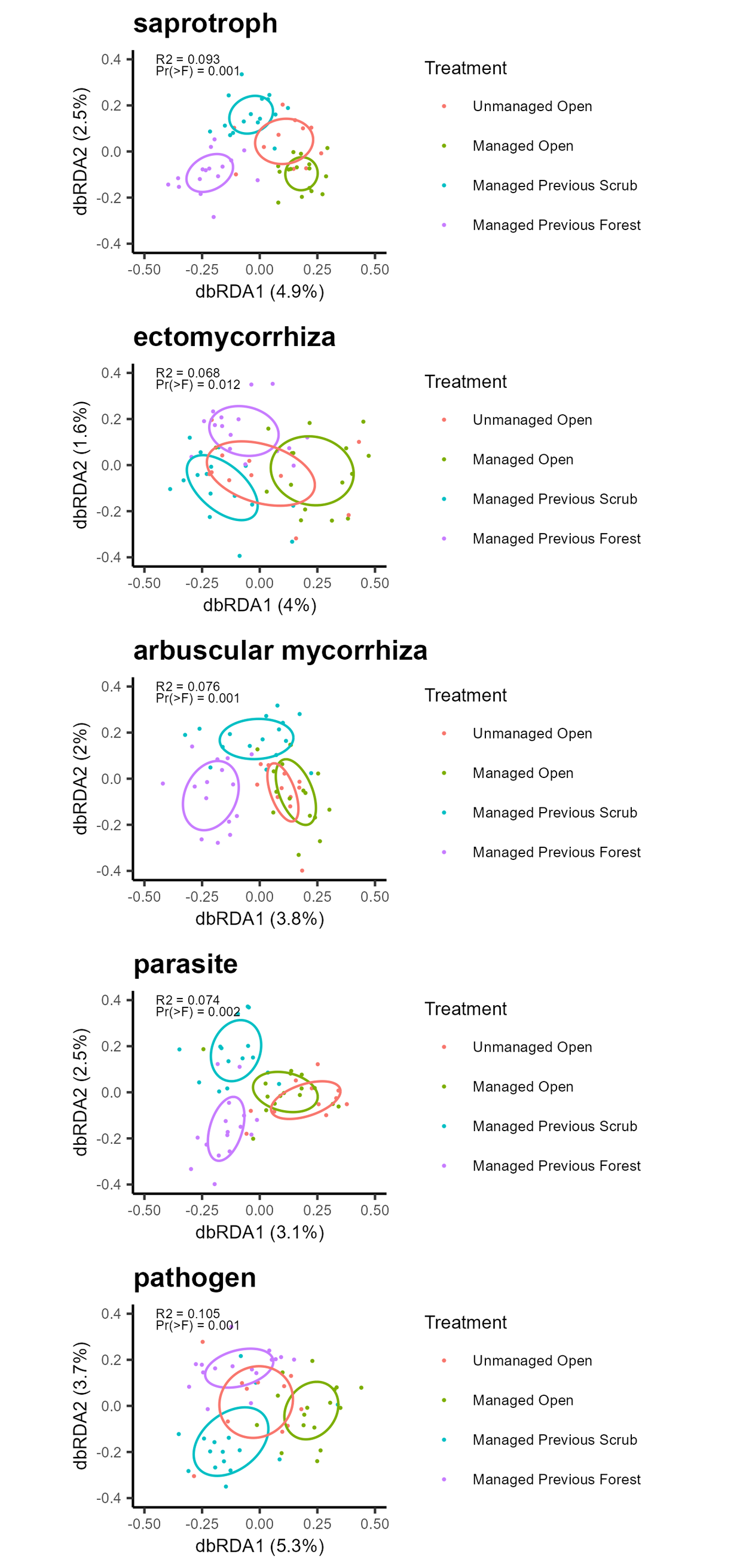

Supplement: S8 Fig — (TIF) [file pone.0292425.s009.tif]

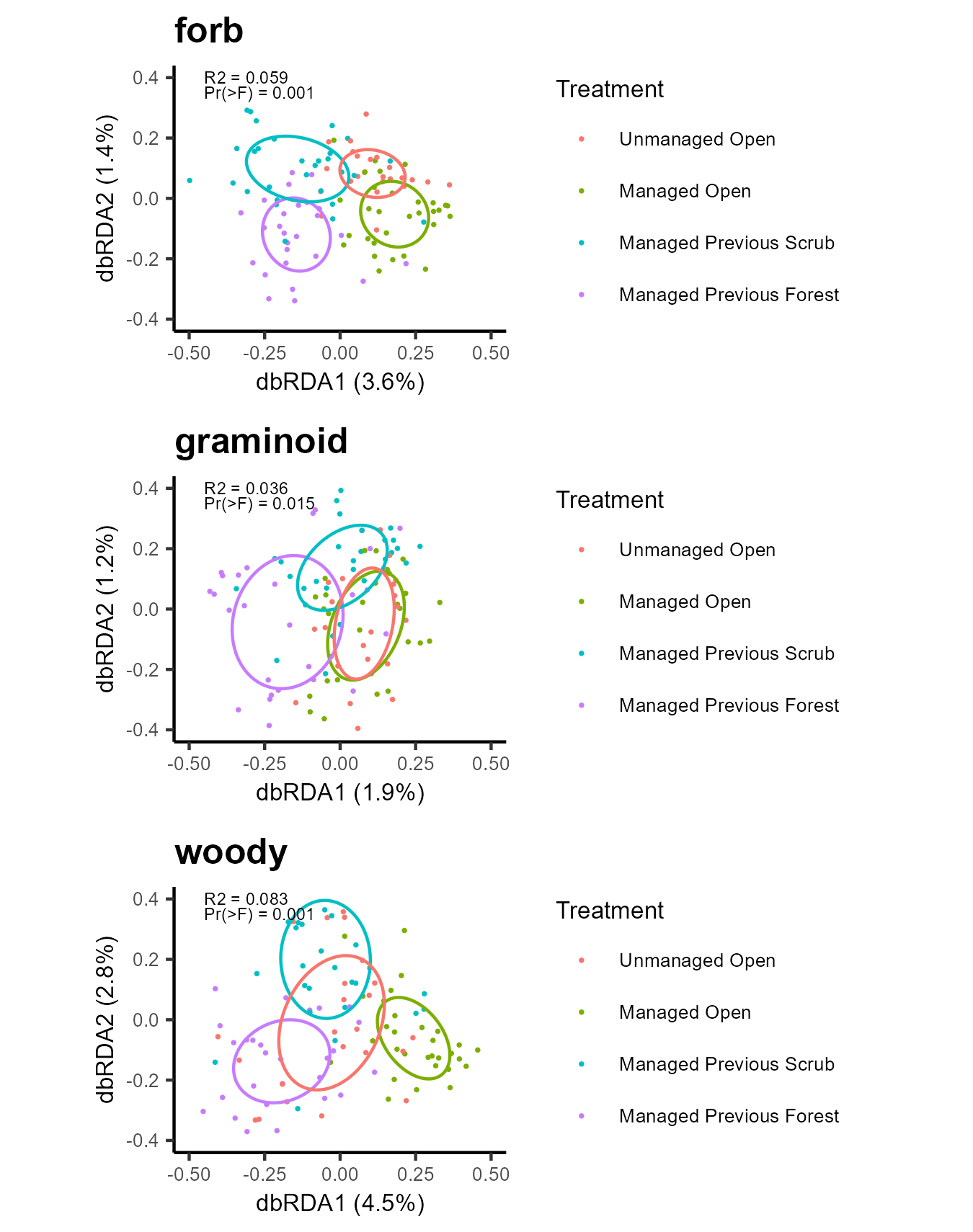

Supplement: S9 Fig — (TIF) [file pone.0292425.s010.tif]
