# Supplementary material for: Soil community composition in dynamic stages of semi-natural calcareous grassland
Source: PLoS One. 2023 Oct 17;18(10):e0292425. doi: 10.1371/journal.pone.0292425 (PMC10581465; doi:10.1371/journal.pone.0292425)
Supplement: Attachment — Submitted filename: Response to Reviewers.docx [file pone.0292425.s011.docx]

PONE-D-23-21042

Soil community composition in dynamic stages of semi-natural calcareous grassland

Dr Jian Liu

Academic Editor

PLOS ONE

Dear Editor,

Thank you very much for sending reviewer comments and an editorial decision on our manuscript. We are extremely grateful to you and the reviewers for the very useful comments. We have considered all of the comments and have now prepared a revised version that we believe is improved and manages to overcome the issues noted by the reviewers.

Please find below a full description of our responses (in regular typeface) to every editor or reviewer comment (in italics).

The academic editor

*In your Methods section, please provide additional information regarding the permits you obtained for the work. Please ensure you have included the full name of the authority that approved the field site access and, if no permits were required, a brief statement explaining why.*

We have added a reference to the permission' Estonian Environmental Board 11.02.2015 nr 17-2.1/15/2415-2' in line number 185 of the manuscript with track changes.

*We note that Figure 1 in your submission contains map/satellite images which may be copyrighted…. We require you to either (a) present written permission from the copyright holder to publish these figures specifically under the CC BY 4.0 license or (b) remove the figures from your submission.*

The map was generated using open-source GIS data. The main plot has data from the Estonian Counties sourced from the Estonian Land Board (<https://geoportaal.maaamet.ee/est/Ruumiandmed/Haldus-ja-asustusjaotus-p119.html>), and the inner map has data sourced from Natural Earth (<https://www.naturalearthdata.com/about/terms-of-use/>). No proprietary or copyrighted data or applications were used to generate the plot. We have added information about the data sources to the legend of Figure 1.

*Please review your reference list to ensure that it is complete and correct*.

Thank you, done.

Reviewer #1:

*Overall the manuscript is well-written and concise. It covers and important topic and should be published after some minor issues are treated and addressed.*

Thank you!

*l56 – "stable states" – what do you mean with this?*

We mean 'the successional stability of ecosystems' and included a more thorough explanation of ecosystem stable states in the Introduction (starting lines 60 of the manuscript with track changes).

*l126-133 The hypotheses are a bit foggy and based on my opinion not very well introduced in the introduction section. The community types studied are not clearly introduced here – it is not clearly said that the effect of restoration works on variously long-lasting encroachment and reference unmanaged grasslands are compared here. In the first hypotheses is not clear what do you mean with "communities vary in parallel"*

We agree with the criticism and have changed the text accordingly. Community types are now clearly introduced (line 148 of the manuscript with track changes). We also rewrote the hypotheses to make them clearer (line 158). We removed the expression 'communities vary in parallel'.

*l338-340 This assumption is rather strong. If something changes faster than the other it does not really means that it driver anything – it could be just simply that this type of community/organism group is more sensitive to the changes than the other. Of course in case of encroachment because of direct competition for light you can imply that the shrub encroachment drives the changes in the herb layer, but this is also highly resistant to a particular level of encroachment. This assumption should be down toned a bit.*

We completely agree and have changed the text accordingly (starting line 410). We basically address which groups of organisms change more quickly and which more slowly. This makes it possible to put forward hypotheses about which groups might drive others, but it does not indicate causal relationships.

Reviewer #2:

*This paper compared variations of soil microbial community composition and vegetation composition following different stages of woody encroachment and analyzed the possible drivers of these variations. The study may provide valuable information for management of semi-natural calcareous grassland.*

Thank you!

*Generally, the manuscript was well written. The introduction was logically organized and the discussion part provided sufficient analysis and interpretation of the data. The following are two suggestions for the authors.*

Thank you!

*（1） The definite conclusions seemed to be missing in both the abstract and in the main text*.

We agree and have now attempted to express the conclusions more clearly.

（*2） In the Methods part, there were no sufficient information on the vegetation (species identity, abundance, canopy height, etc.), climate, and soil etc. for each site sampled.*

We appreciate this observation and have added more descriptive information about the study sites.

Thank you once again for providing such a thoughtful and thorough review of our work. We look forward to hearing from you.

Yours sincerely, on behalf of the authors

Ayesh Wipulasena
